# Glioma infiltration of the corpus callosum: early signs detected by DTI

**DOI:** 10.1007/s11060-013-1049-y

**Published:** 2013-04-01

**Authors:** K. Kallenberg, T. Goldmann, J. Menke, H. Strik, H. C. Bock, F. Stockhammer, J. H. Buhk, J. Frahm, P. Dechent, M. Knauth

**Affiliations:** 1 Neuroradiology, Universitätsmedizin, Georg-August-University Göttingen, Robert-Koch-Str. 40, 37099 Göttingen, Germany; 2 MR-Research in Neurology and Psychiatry, Universitätsmedizin, Georg-August-University Göttingen, Robert-Koch-Str. 40, 37099 Göttingen, Germany; 3 Radiology, Universitätsmedizin, Georg-August-University Göttingen, Göttingen, Germany; 4 Neurology, University Medical Center, Philipps University Marburg, Marburg, Germany; 5 Neurosurgery, Universitätsmedizin, Georg-August-University Göttingen, Göttingen, Germany; 6 Neuroradiology, University Medical Center Hamburg-Eppendorf, Hamburg, Germany; 7 Biomedizinische NMR Forschungs GmbH am MPI für biophysikalische Chemie, Göttingen, Germany

**Keywords:** Glioma, Corpus callosum, Magnetic resonance imaging, Diffusion tensor imaging

## Abstract

The most frequent primary brain tumors, anaplastic astrocytomas (AA) and glioblastomas (GBM): tend to invasion of the surrounding brain. Histopathological studies found malignant cells in macroscopically unsuspicious brain parenchyma remote from the primary tumor, even affecting the contralateral hemisphere. In early stages, diffuse interneural infiltration with changes of the apparent diffusion coefficient (ADC) and fractional anisotropy (FA) is suspected. The purpose of this study was to investigate the value of DTI as a possible instrument of depicting evidence of tumor invasion into the corpus callosum (CC). Preoperatively, 31 patients with high-grade brain tumors (8 AA and 23 GBM) were examined by MRI at 3 T, applying a high-resolution diffusion tensor imaging (DTI) sequence. ADC- and FA-values were analyzed in the tumor-associated area of the CC as identified by fiber tracking, and were compared to matched healthy controls. In (MR-)morphologically normal appearing CC the ADC values were elevated in the tumor patients (*n* = 22; 0.978 × 10^−3^ mm²/s) compared to matched controls (0.917 × 10^−3^ mm²/s, *p* < 0.05), and the corresponding relative FA was reduced (rFA: 88 %, *p* < 0.01). The effect was pronounced in case of affection of the CC visible on MRI (*n* = 9; 0.978 × 10^−3^ mm²/s, *p* < 0.05; rFA: 72 %, *p* < 0.01). Changes in diffusivity and anisotropy in the CC can be interpreted as an indicator of tumor spread into the contralateral hemisphere not visible on conventional MRI.

## Introduction

Magnetic resonance imaging (MRI) plays a central role in the staging and treatment of patients with brain tumors. While conventional MRI with T2, FLAIR, and contrast-enhanced T1-weighted sequences unravel the size, shape, and structure of lesions, the use of perfusion- and diffusion-weighted MRI adds information on the microstructural architecture and regional blood flow.

The treatment of anaplastic astrocytomas and glioblastomas first comprises a maximum surgical resection [1], which is to be followed by radiation and chemotherapy. Although affected patients often show only a small tumor burden and advanced imaging methods contribute to an adequate diagnosis and tumor localization, higher-grade astrocytomas are still considered incurable even when well accessible for surgical and radiotherapeutic treatment [2]. The palliative treatment aims at maintaining the quality of life with smallest neurological-cognitive deficits as long as possible [3]. However, in these patients the median survival time is less than 2 years, and despite of general advances in diagnosis and therapy, this poor prognosis has improved only marginally during the past 30 years [4, 5].

Histopathological studies have reported the infiltrative growth of glioblastoma multiforme (GBM) cells in apparently unaffected brain regions remote from the confirmed primary lesion [6–9] without correlating changes on computed tomographic or MR images [10, 11]. Localized proton MR spectroscopy (MRS) gives insights into tissue composition and intracellular metabolism and already provides subtle hints about remote infiltration by GBM cells [12–14]. In early tumor stages, diffuse interneural infiltration with changes of the apparent diffusion coefficient (ADC) and fractional anisotropy (FA) is suspected. Pertinent alterations were indeed observed by diffusion tensor imaging (DTI) and/or MRS of normal appearing white matter in the hemisphere contralateral to the actual glioma [12, 13].

In analogy to a previous case report [15] the purpose of this study was to investigate the value of DTI as a possible instrument of depicting evidence of tumor invasion into the corpus callosum (CC).

## Materials and methods

### Patients

The local ethics committee approved this work according to the declaration of Helsinki. Written informed consent was obtained from all patients and control subjects. Over a time period of 2 years pre-operative DTI was prospectively performed in all consecutive cases of suspected brain tumor before treatment. Only patients with post-operative definite histopathological confirmation of a high grade brain tumor (anaplastic astrocytoma WHO III° or glioblastoma WHO IV°) were included. Healthy age- and sex-matched volunteers without any relevant medical history served as normal controls. Data analyses were performed retrospectively.

### MRI

All studies were performed on the same 3T MR system (Magnetom Trio, Siemens Medical Solutions, Erlangen/Germany). Anatomical imaging included three-dimensional (3D) T1-weighted (T1w) fast low-angle shot (FLASH) MRI (time to repetition TR 11 ms; time to echo TE 4.9 ms; flip angle 15°; isotropic image resolution 1 × 1 × 1 mm^3^). Diffusion tensor imaging employed a single-shot stimulated echo acquisition mode (STEAM) sequence with 24 different diffusion gradient directions (*b*-value 1,000 s/mm², 38 axial sections, 3 acquisitions) at 2.2 × 2.2 × 2.2 mm³ image resolution. For further details see [15].

### Image analysis

The image analyses were performed retrospectively. During post-processing the diffusion-weighted images were smoothed with a 3D Gaussian filter (*σ* = 2.2 mm). The diffusion tensor was then calculated by a linear least-squares algorithm using an in-house software (DeffCon [16]). The tumor area was determined utilizing the clinical MR study comprising FLAIR, T2, T1 before and after gadolinium injection as well as perfusion and diffusion weighted images and manually defined on the 3D T1w datasets, and served as starting point for a fiber tracking algorithm. The fiber tracts crossing the CC were utilized to determine the region of the CC for the FA and ADC analysis. In detail, fiber assignments were estimated from the 3D FA values by a continuous axonal projection tracking algorithm (FACT [17]). This algorithm terminated the tracking of an individual fiber at FA-values below 0.15, or if the local main diffusion tensor direction deviated more than 40° in consecutive tracking steps [16]. In every subject, average FA- and ADC-values were quantified from the midsagittal plane and both adjacent slices in the affected parts of the CC, as localized by tractography.

To account for differences in absolute anisotropy in the CC sections [16] and to facilitate group comparisons irrespective of the CC section affected, the FA data were normalized by correlating to the individual FA-values of the accordant CC section of the age- and sex-matched control, referred to as *relative fractional anisotropy* (rFA).

### Statistical analyses

Three groups were established according to the comprehensive analysis of the clinical MR study performed by two experienced neuroradiologists in consensus and, additional the medical history for group 3:

- 1. Tumor patients with obvious affection of the CC on MRI (CC-affect),
- 2. Tumor patients without obvious affection of the CC on MRI (CC-normal),
- 3. Age- and sex-matched healthy subjects in a control group (control).

The ADC- and rFA-values from the patients and subjects were analyzed by a paired student’s *t* test. The patients “CC-affect” and “CC-normal” were analyzed by an unpaired student’s *t* test.

A survival analysis was performed by applying a log-rank (Mantel-Cox) test, comparing the five patients with the highest ADC-values to the five patients with the lowest ADC-values. *p*-values <0.05 were regarded as significant.

## Results

### Demographic data

31 patients fulfilled the inclusion criteria and completed the DTI measurement: 12 females, 19 males; mean age 56.9 years, range [22–77 years]. The post-operative neuropathological diagnosis was anaplastic astrocytoma (WHO III°) in 8 cases and glioblastoma multiforme (WHO IV°) in 23 cases. DTI datasets of healthy age- and sex-matched volunteers served as control (mean age 58.7 years, range [23–81 years]).

### Apparent diffusion coefficient

In the control subjects the ADC-values showed a non-significant trend to increase with age (*r* = 0.31, *p* = 0.14). Compared to the healthy controls, the ADC-values in the tumor patients rendered more inhomogeneous results, specifically if the CC was affected according to MRI (CC-affect). The patients’ ADC-values were significantly higher relative to controls (*p* < 0.05) (Table 1). Within the patient group, the presence of CC infiltration had no effect on the ADC-values, i.e. the average ADC-values in CC-affect or CC-normal patients were almost similar (0.97769 × 10^−3^ vs 0.97786 × 10^−3^ mm²/s; *p* = 0.498).

**Table 1:** ADC-values in the corpus callosum associated to the glioma location compared to healthy age- and sex-matched controls

	ADC (95 % CI) (×10^−3^ mm²/s)	*p*
Controls (*n* = 31)	0.917 (0.893;0.941)	
Patients (CC-normal) (*n* = 22)	0.978 (0.920;1.036)	<0.05 (vs control)
Patients (CC-affect) (*n* = 9)	0.978 (0.911;1.044)	<0.05 (vs control)

95 % CI = 95 % confidence interval

### Relative fractional anisotropy

Regarding normal controls and the CC areas of patients not related to the tumor FA values significantly decreased with age (*r* = −0.59, *p* < 0.01).

**Fig. 1:**
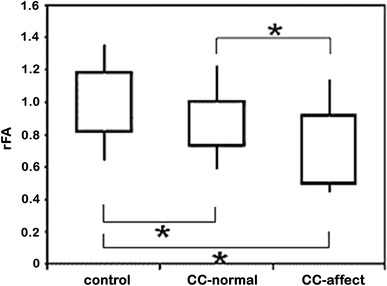
*Boxplot* of rFA values of patients with (CC-affect) and without (CC-normal) obvious affection of the corpus callosum and healthy controls demonstrating significant *asterisk* differences between the two patient groups and controls as well as between the patient groups

There was a significant rFA reduction in the tumor corresponding region of the CC of both patient groups compared to controls (Table 2) and comparing the patients with CC affection to those with inconspicuous CC (Fig. 1).

**Table 2:** Relative FA (rFA) values in the corpus callosum associated to the glioma location with (CC-affect) and without (CC-normal) obvious affection of the CC compared to healthy age- and sex-matched controls

	rFA (95 % CI)	*p*
Controls (*n* = 31)	1.000 (0.959;1.041)	
Patients (CC-normal) (*n* = 22)	0.880 (0.816;0.944)	<0.01(vs control)
Patients (CC-affect) (*n* = 9)	0.721 (0.605;0.838)	<0.0001(vs control); <0.01 (vs CC-normal)

### Survival data

**Fig. 2:**
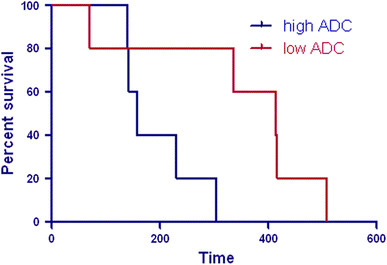
The Kaplan–Meier-curve comparing the five patients with the lowest ADC-values to the five patients with highest ADC-values and without visible CC infiltration demonstrates a longer survival with low or normal ADC-values

There was no significant difference between patients with CC infiltration compared to those without (median survival time: CC-affect 335.0 days vs CC-normal 358.5 days; *p* > 0.05). However, among the patients without CC infiltration the five patients with the lowest ADC-values tended to live longer than the five patients with the highest ADC-values (Fig. 2); median survival time: “ADC high” 157.0 days, “ADC low” 413.0 days; *p* < 0.05. There was no significant difference between the two groups regarding tumor size, resection or therapy.

### Illustrative case

**Fig. 3:**
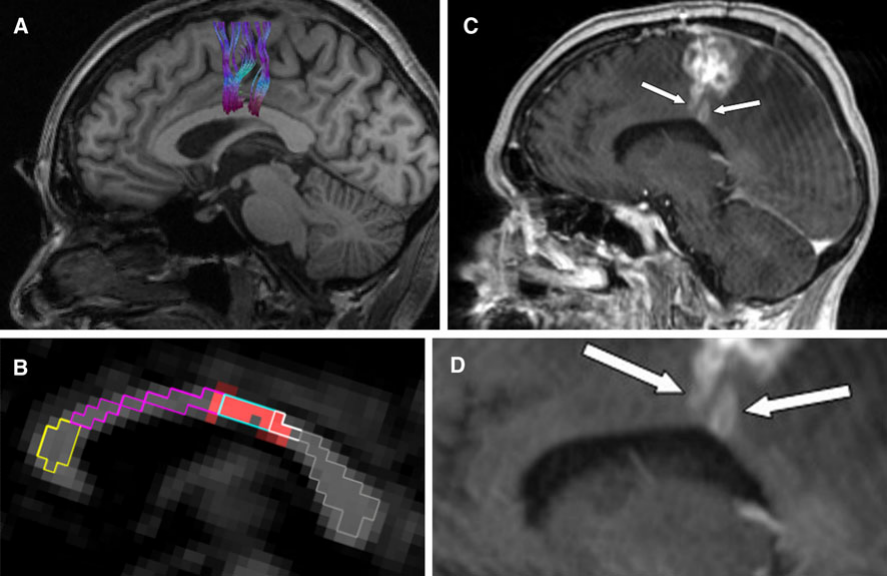
The 3D-fiber tracts are projected on the sagittal midline 2D-image from the T1 dataset demonstrating the localization of the passage of the CC (**a**). The fiber tracts originating from a glioblastoma crossing the truncus of the CC—marked in *solid red* (**b**)—identify an area mainly in region 2 [16] marked in *light blue* (**b**)—Without any abnormal signal or contrast enhancement (**c**). The follow-up examination 12 months later reveals recurrent tumor infiltrating of the CC with contrast enhancement in the previously identified area (**d**)

In order to illustrate possible infiltration patterns of malignant brain tumors, Fig. 3 shows pre-operative and follow-up T1-weighted MRI of a 65 year-old female with newly diagnosed GBM. At first, tumor growth was limited to the right hemisphere and fiber-tracking revealed tumor-associated nerve fibers crossing the trunk of the CC as shown below. With the tumor growth progressing despite surgery and concomitant radio-chemotherapy, post-operative follow-up examination displayed enhancement of contrast agent in those regions of the CC which showed to contain fibers connecting the primary focus with the contralateral hemisphere 10 month before.

## Discussion

Only about 2 % of patients with high-grade brain tumors survive the first 5 years after diagnosis [18]. One of the main factors for the poor prognosis of glioblastomas is the invasiveness of malignant cells [19]. Histopathological studies showed glioma cells remote [6, 10] from the histologically identical main tumor bulk [9] only to differ in growth rate. These infiltrating glioma cells can hardly be imaged by standard techniques [11]. However, diffusion and metabolic abnormalities detected by DWI/DTI and proton MRS were found outside the borders of malignant brain tumors defined by conventional MRI as potential indicators of tumor infiltration or malignant transformation [12, 13, 20].

A number of effects may account for the increase of ADC-values in the section of the CC corresponding to the tumor location: An interaction of tumor cells with the extracellular matrix (ECM) is mandatory to enable cell migration. The invasiveness depends on destruction of ECM components as well as on penetration between adjacent normal brain structures [21]. Glioma cells initially infiltrate or spread micro-invasively between and around neurons and penetrate into the fiber tracts of white matter [22], causing a local displacement of the normal parenchyma without neuronal damage [23]. Then the local brain tissue is untightened and displaced under the influence of proteases [24]. Furthermore, the growth of a tumor in the CNS is associated with a disturbance of the blood–brain barrier, which leads to a vasogenic edema and an increase in parenchymal water content, which in turn increases the extracellular space and the corresponding ADC-value [25].

While disease advances, nerve cells may be destroyed and replaced by tumor cells [22]. This results in a decrease of ADC-values [26] and explains the ADC inhomogeneities among cases where MRI depicted infiltration of the CC. Regarding fractional anisotropy, a negative correlation between FA values versus cellular density and rate of tumor infiltration has been reported [27, 28], corresponding to our findings.

Abnormal FA- or ADC-values support [29] but do not prove glioma invasion since ADC cannot definitely differentiate between tumor and edema [30]. Corresponding to our data, however: tumor recurrence frequently occurs in areas of previously enhanced diffusivity, especially if fiber tracking suggests a connection to the tumor [31]. Unfortunately, only a small number of patients survived long enough to be monitored for consecutive tumor infiltration into the CC.

### Limitations

Age- and sex-matched healthy individuals served as controls to minimize potential influences on DTI values [32, 33].

According to ethical considerations no specimen from normal appearing parenchyma of the corpus callosum was collected for histopathological analysis.

## Conclusion

In patients with malignant gliomas brain tumors, analyzing the CC which connects both hemispheres is of major importance, since its infiltration indicates tumor spread into the contralateral hemisphere which in turn downgrades the prognosis. Our data suggest that changes in ADC and in FA in the tumor corresponding areas of the CC may be an early indicator for tumor cell infiltration in those areas. Larger clinical follow-up studies are required to confirm this postulation, because it may have a large impact on prognosis and therapy.
